# Bonobo and chimpanzee gestures overlap extensively in meaning

**DOI:** 10.1371/journal.pbio.2004825

**Published:** 2018-02-27

**Authors:** Kirsty E. Graham, Catherine Hobaiter, James Ounsley, Takeshi Furuichi, Richard W. Byrne

**Affiliations:** 1 School of Psychology and Neuroscience, University of St Andrews, St Andrews, United Kingdom; 2 Department of Psychology, University of York, York, United Kingdom; 3 School of Biology, University of St Andrews, St Andrews, United Kingdom; 4 Primate Research Institute, Kyoto University, Inuyama, Japan; Queen Mary University of London, United Kingdom of Great Britain and Northern Ireland

## Abstract

Cross-species comparison of great ape gesturing has so far been limited to the physical form of gestures in the repertoire, without questioning whether gestures share the same meanings. Researchers have recently catalogued the meanings of chimpanzee gestures, but little is known about the gesture meanings of our other closest living relative, the bonobo. The bonobo gestural repertoire overlaps by approximately 90% with that of the chimpanzee, but such overlap might not extend to meanings. Here, we first determine the meanings of bonobo gestures by analysing the outcomes of gesturing that apparently satisfy the signaller. Around half of bonobo gestures have a single meaning, while half are more ambiguous. Moreover, all but 1 gesture type have distinct meanings, achieving a different distribution of intended meanings to the average distribution for all gesture types. We then employ a randomisation procedure in a novel way to test the likelihood that the observed between-species overlap in the assignment of meanings to gestures would arise by chance under a set of different constraints. We compare a matrix of the meanings of bonobo gestures with a matrix for those of chimpanzees against 10,000 randomised iterations of matrices constrained to the original data at 4 different levels. We find that the similarity between the 2 species is much greater than would be expected by chance. Bonobos and chimpanzees share not only the physical form of the gestures but also many gesture meanings.

## Author summary

Bonobos and chimpanzees are closely related members of the great ape family, and both species use gestures to communicate. We are able to deduce the meaning of great ape gestures by looking at the ‘Apparently Satisfactory Outcome’ (ASO), which reflects how the recipient of the gesture reacts and whether their reaction satisfies the signaller; satisfaction is shown by the signaller ceasing to produce more gestures. Here, we use ASOs to define the meaning of bonobo gestures, most of which are used to start or stop social interactions such as grooming, travelling, or sex. We then compare the meanings of bonobo gestures with those of chimpanzees and find that many of the gestures share the same meanings. Bonobos and chimpanzees could, in principle, understand one another’s gestures; however, more research is necessary to determine how such gestures and gesture meanings are acquired.

## Introduction

In a series of well-known children’s books, Doctor Dolittle was able to talk to nonhuman animals, but in reality, deciphering meaning in nonhuman communication presents a much bigger challenge. First, there is the question of whether animal signals can be said to have ‘meanings’ or merely ‘functions’. Functions are known for many animal signals: for example, various species are able to decode complex information from their conspecifics’ calls on the location or class of food or predators [1–4], level of risk [4,5], and size of predator [6]. However, for meaning, a signal needs to be produced intentionally—the signaller must aim to change the behaviour (first-order intentional) or the mental state (at least second-order intentional) of the recipient [7–9].

Mounting evidence shows that, unlike most nonhuman animals [10], great apes habitually engage in first-order intentional communication: great apes routinely direct their gestures towards a specific recipient; monitor that recipient’s attentional state and choose gestures appropriate to it; wait for the recipient to respond; and, if the recipient does not respond, they persist and elaborate with further gestures [11–17]. These criteria demonstrate that the signaller has a specific outcome in mind and uses gestures to achieve that outcome [18]. It has also been argued that to have meaning, communication needs to be ostensive, drawing attention to the fact that it is being used to communicate [19]. In developmental psychology, eye gaze is taken as an ostensive cue; the audience checking performed by great apes before gesturing serves the same ostensive function [20]. Because great apes deploy gestures intentionally, it is appropriate to go beyond simply describing their function and enquire about the intended meaning that a signaller aims to achieve by gesturing [20]. Although we focus on gestural communication, it should be noted that great apes also appear to deploy some vocal signals intentionally [21–23]. Moreover, we focus on a Gricean approach to meaning, rather than a semantic approach [24,25], given that few great ape gestures appear to be referential (but see [26]).

The second challenge is that gesture meanings must be deduced indirectly. Past studies have tackled the issue of meaning by looking at the context in which gestures occur [16,27], thereby showing that the same gesture may occur in several contexts. We have taken a different approach. By using the reaction that each gesture elicits, but only in cases where the signaller’s behaviour indicates that this reaction was their intended aim, we hope to pin down the signaller’s intended meaning for each specific gesture. The meaning of a gesture can thus be defined by the ‘Apparently Satisfactory Outcome’ (ASO), the reaction of the recipient that satisfies the signaller as shown by cessation of gesturing [28]. This method indicates the individual signaller’s intended meaning in each instance, and across many instances and individuals, one can examine the gesture’s general meaning(s) in a population. Aggregating the meanings represents population level patterns of meaning but does not infer that meanings are conventionalised nor indeed does it imply any particular ontogeny for gesture meanings.

Defining meaning by ASOs, wild chimpanzees use their gestures to achieve at least 19 ASOs; that is, their gestures achieve 19 types of behavioural response from the recipient [28]. Each gesture type has a distinct (set of) meaning(s) that is calculated by comparing the distribution of meanings for a gesture type to the distribution of meanings across all gesture types [28]. Using ASOs to define the meaning of gestures is a relatively new approach, so gesture meanings have not yet been defined for our other closest living relative—the bonobo (or ‘bilia’, as the species is locally known) [29]. Our study is the first to investigate meaning in the natural gestural repertoire of wild bonobos.

Once the meanings of bonobo gestures are defined, we can examine the gestural overlap of bonobos and chimpanzees. All species of nonhuman great ape share the majority of their gestural repertoire in terms of the gestures’ physical forms. The overlap for chimpanzees and bonobos is 88%–96% [30]; for chimpanzees and gorillas, 60% [31]; and for chimpanzees and orangutans, 80% [31]. But simply using the same actions does not mean that chimpanzees and bonobos share a communication system (that is, that a chimpanzee and bonobo would in principle be able to understand one another). Only if bonobo and chimpanzee gestures share the same meanings can they be said to share the same system of communication.

Deciding that issue is not straightforward. Ape gestural repertoires are large, with over 70 distinct gestures in the chimpanzee and bonobo catalogues. In captivity, large quantities of gestural data can be collected very quickly, but the majority of it occurs during play [32,33]. Data from the wild are needed to examine the full breadth of meaning expressed in nonplayful ape communication. Previous studies have used traditional analyses of variance or goodness of fit tests, demonstrating that different individuals within a chimpanzee group use the same gesture to achieve the same outcome [28]. However, despite data sets containing thousands of gesture cases, large repertoires and the regular use of only a subset of these gestures [34] limits the number of gesture types that can be examined in this way. Furthermore, those tests are not suited to data sets in which many of the possible outcomes never occur for each signal type, as we would expect in a system of communication in which specific signals are employed for specific outcomes. We have therefore adapted methods from numerical ecology to compare the similarity between the meanings of bonobo and chimpanzee gestures. In doing so, we offer the first analysis that examines whether the overlap in the physical form of bonobo and chimpanzee gestures extends to their meaning.

## Results

### Bonobo gesture meanings

**Fig 1:**
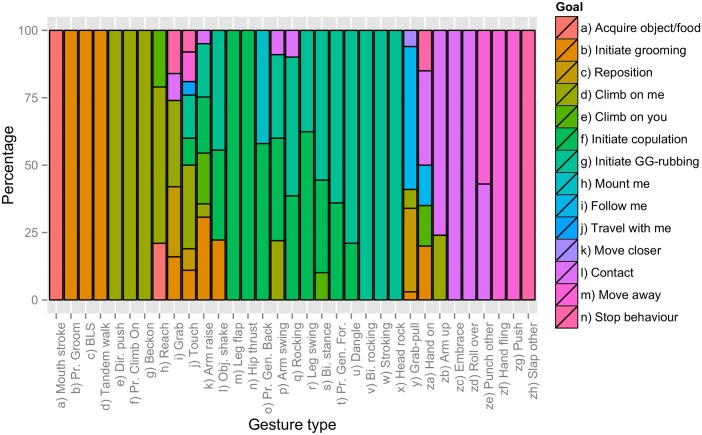
Proportional stacked histogram for ASOs achieved by each gesture type (values from S1 Table). ASOs are coloured in a gradient adjacent to similar ASOs, and gesture types are arranged adjacent to those with similar profiles. ASO distributions for chimpanzee gestures were reported in [28]. ASO, Apparently Satisfactory Outcome.

We analysed 2,321 intentional gesture instances (occasions on which a gesture was used) that successfully achieved an ASO. These instances concerned 33 gesture types (categories of gestures that share the same physical form) [30,31] (S1 Table) and 14 different ASOs: ‘Acquire object/food’, ‘Climb on me’, ‘Climb on you’, ‘Contact’, ‘Follow me’, ‘Initiate grooming’, ‘Mount me’, ‘Move closer’, ‘Reposition’, ‘Initiate copulation’, ‘Initiate genito-genital rubbing (GG-rubbing)’, ‘Travel with me’, ‘Move away’, and ‘Stop behaviour’. The first 12 of these ASOs served to initiate or develop an activity, and the last 2 served to stop an activity. Of the 33 gesture types, 17 had only a single ASO, 6 had 2 ASOs, and 10 had >2 ASOs (Fig 1). The mean number of ASOs per gesture type was 2.27 ± 1.84 (median = 2, range 1–8).

**Fig 2:**
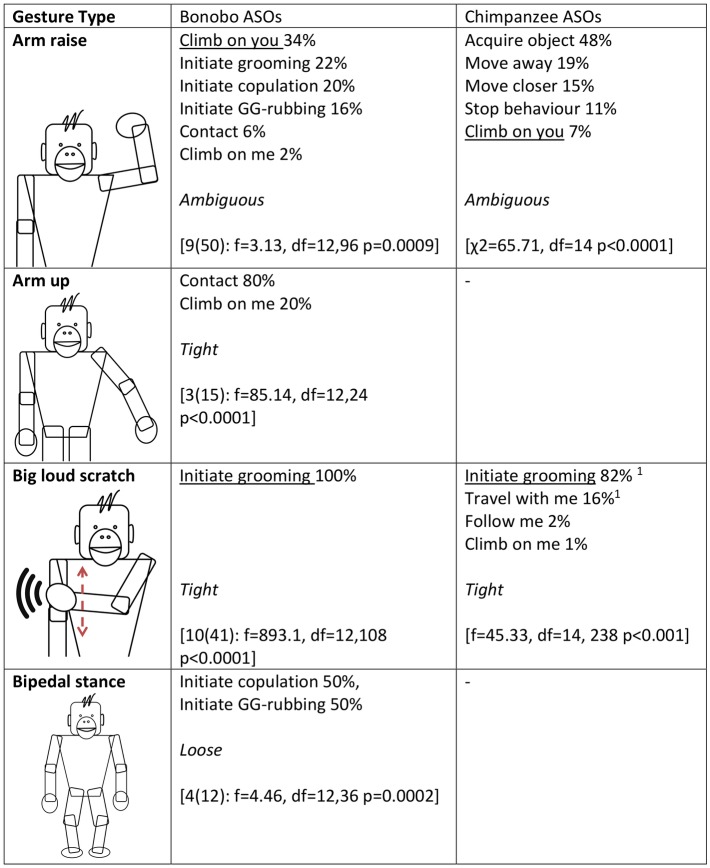
Gesture types that were analysed for ASO distribution (video examples for all gesture types can be found at http://greatapedictionary.wp.st-andrews.ac.uk/video-resources/gesture-videos/). All ASOs are given for each gesture type, in descending order from most to least frequent, as a percentage of all instances for all ASOs included in analysis (values in S1 Table, raw data in S1 Data). For bonobos, results for ANOVA are given in square brackets, e.g., [N(n): ANOVA results], with N as number of individuals and n as number of gesture instances (for age and sex of contributing individuals, see S2 Table); a significant effect shows that gesture usage differs from the average distribution of gesture frequencies. For chimpanzees, ANOVA or chi-squared analyses were performed [28]; square brackets contain published results. Underlined ASOs are shared by both chimpanzees and bonobos for that gesture type. This chi-squared analysis was conducted in Hobaiter & Byrne 2014 after checking and finding no effect of signaller identity on gestural meaning. We have included it for comparison but recognise that chi-squared analyses risk pseudoreplication. For analyses, we combined several ASOs from [28]: ‘Initiate copulation’ includes ‘Sexual attention—female’ and ‘Sexual attention—male’; ‘Initiate grooming’ includes ‘initiate grooming’ and ‘direct attention’; ‘Travel with me’ includes ‘Travel with me (adult)’ and ‘Travel with me (young)’. ASO, Apparently Satisfactory Outcome.

**Fig 3:**
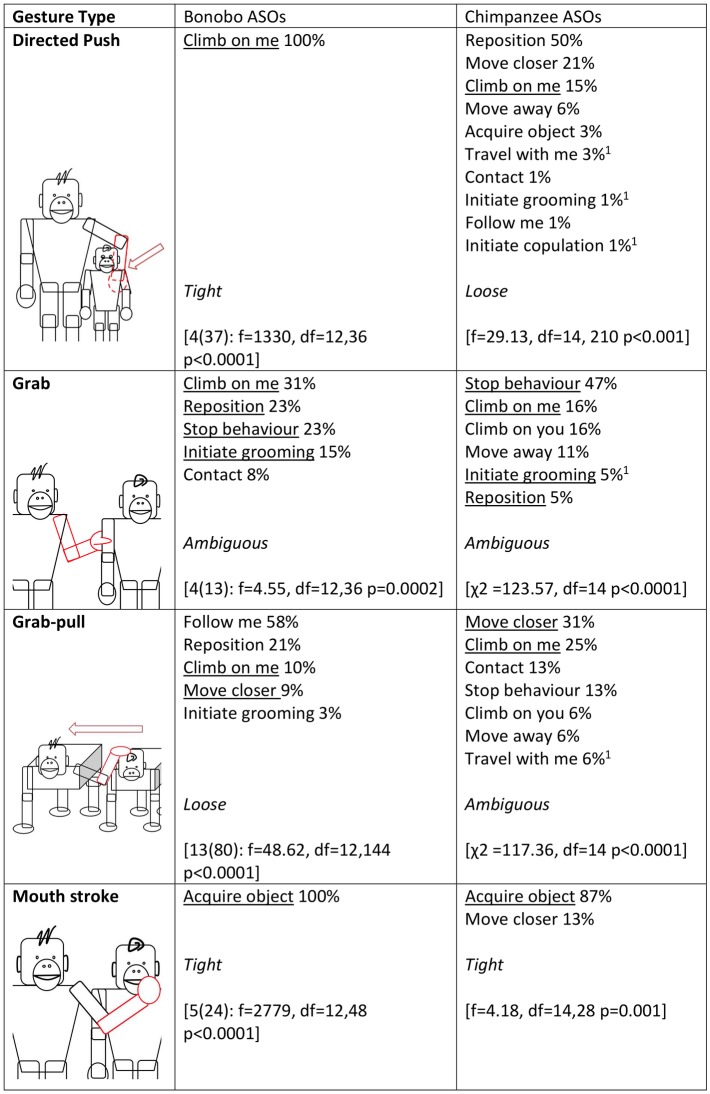
Gesture types that were analysed for ASO distribution (video examples for all gesture types can be found at http://greatapedictionary.wp.st-andrews.ac.uk/video-resources/gesture-videos/). All ASOs are given for each gesture type, in descending order from most to least frequent, as a percentage of all instances for all ASOs included in analysis (values in S1 Table, raw data in S1 Data). For bonobos, results for ANOVA are given in square brackets, e.g., [N(n): ANOVA results], with N as number of individuals and n as number of gesture instances (for age and sex of contributing individuals, see S2 Table); a significant effect shows that gesture usage differs from the average distribution of gesture frequencies. For chimpanzees, ANOVA or chi-squared analyses were performed [28]; square brackets contain published results. Underlined ASOs are shared by both chimpanzees and bonobos for that gesture type. This chi-squared analysis was conducted in Hobaiter & Byrne 2014 after checking and finding no effect of signaller identity on gestural meaning. We have included it for comparison but recognise that chi-squared analyses risk pseudoreplication. For analyses, we combined several ASOs from [28]: ‘Initiate copulation’ includes ‘Sexual attention—female’ and ‘Sexual attention—male’; ‘Initiate grooming’ includes ‘initiate grooming’ and ‘direct attention’; ‘Travel with me’ includes ‘Travel with me (adult)’ and ‘Travel with me (young)’. ASO, Apparently Satisfactory Outcome.

**Fig 4:**
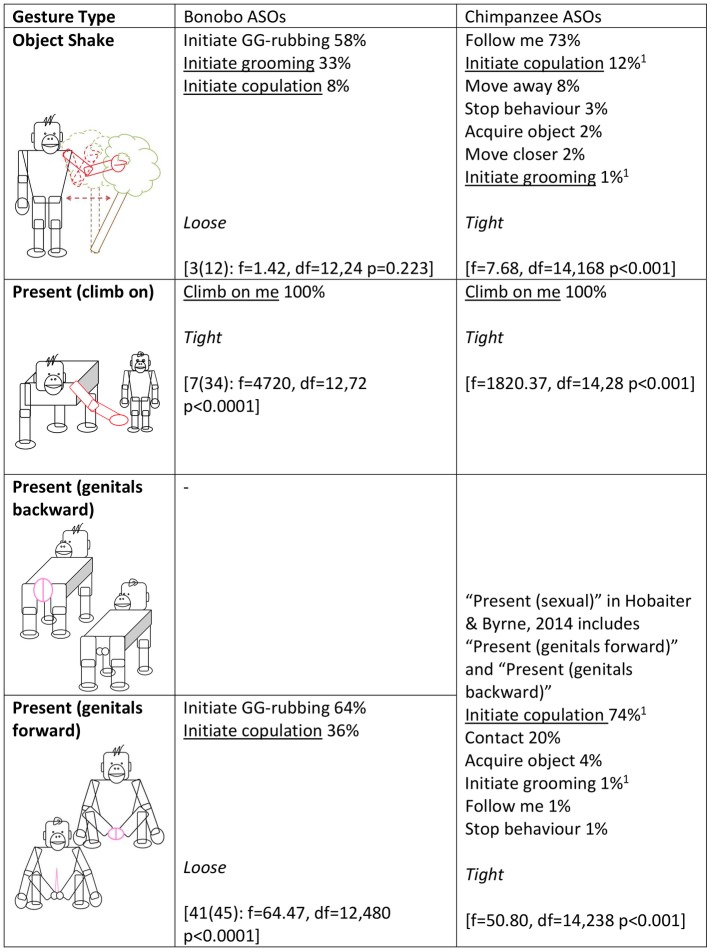
Gesture types that were analysed for ASO distribution (video examples for all gesture types can be found at http://greatapedictionary.wp.st-andrews.ac.uk/video-resources/gesture-videos/). All ASOs are given for each gesture type, in descending order from most to least frequent, as a percentage of all instances for all ASOs included in analysis (values in S1 Table, raw data in S1 Data). For bonobos, results for ANOVA are given in square brackets, e.g., [N(n): ANOVA results], with N as number of individuals and n as number of gesture instances (for age and sex of contributing individuals, see S2 Table); a significant effect shows that gesture usage differs from the average distribution of gesture frequencies. For chimpanzees, ANOVA or chi-squared analyses were performed [28]; square brackets contain published results. Underlined ASOs are shared by both chimpanzees and bonobos for that gesture type. This chi-squared analysis was conducted in Hobaiter & Byrne 2014 after checking and finding no effect of signaller identity on gestural meaning. We have included it for comparison but recognise that chi-squared analyses risk pseudoreplication. For analyses, we combined several ASOs from [28]: ‘Initiate copulation’ includes ‘Sexual attention—female’ and ‘Sexual attention—male’; ‘Initiate grooming’ includes ‘initiate grooming’ and ‘direct attention’; ‘Travel with me’ includes ‘Travel with me (adult)’ and ‘Travel with me (young)’. ASO, Apparently Satisfactory Outcome.

**Fig 5:**
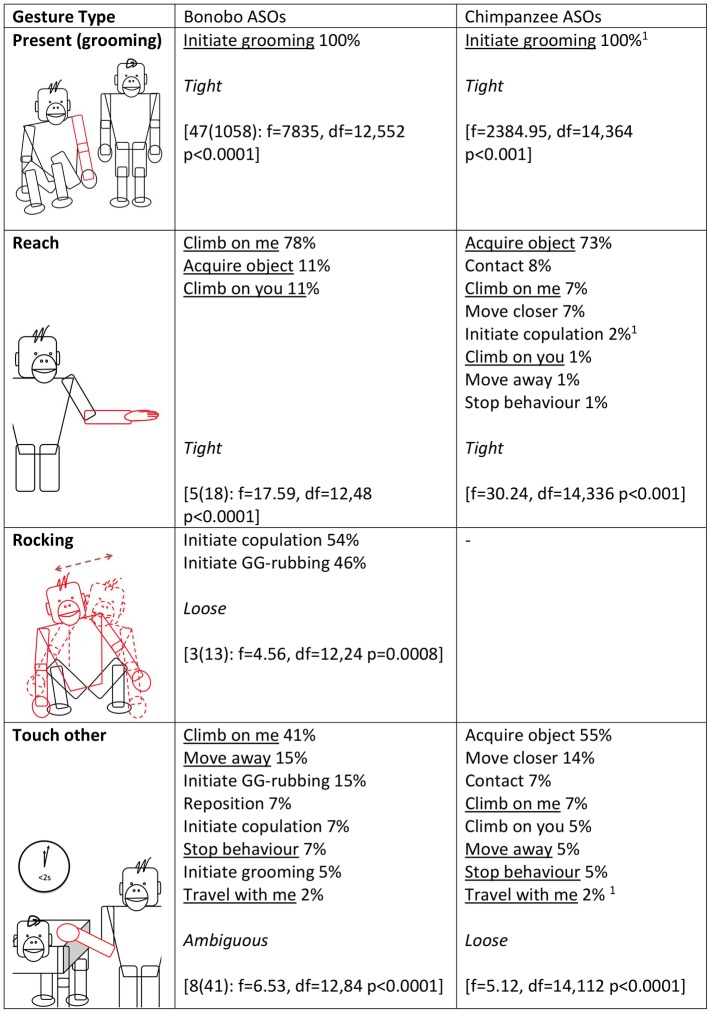
Gesture types that were analysed for ASO distribution (video examples for all gesture types can be found at http://greatapedictionary.wp.st-andrews.ac.uk/video-resources/gesture-videos/). All ASOs are given for each gesture type, in descending order from most to least frequent, as a percentage of all instances for all ASOs included in analysis (values in S1 Table, raw data in S1 Data). For bonobos, results for ANOVA are given in square brackets, e.g., [N(n): ANOVA results], with N as number of individuals and n as number of gesture instances (for age and sex of contributing individuals, see S2 Table); a significant effect shows that gesture usage differs from the average distribution of gesture frequencies. For chimpanzees, ANOVA or chi-squared analyses were performed [28]; square brackets contain published results. Underlined ASOs are shared by both chimpanzees and bonobos for that gesture type. This chi-squared analysis was conducted in Hobaiter & Byrne 2014 after checking and finding no effect of signaller identity on gestural meaning. We have included it for comparison but recognise that chi-squared analyses risk pseudoreplication. For analyses, we combined several ASOs from [28]: ‘Initiate copulation’ includes ‘Sexual attention—female’ and ‘Sexual attention—male’; ‘Initiate grooming’ includes ‘initiate grooming’ and ‘direct attention’; ‘Travel with me’ includes ‘Travel with me (adult)’ and ‘Travel with me (young)’. ASO, Apparently Satisfactory Outcome.

Next, in accordance with [28], we used a series of ANOVAs to analyse whether the distribution of ASOs for a given gesture type differed from the average distribution of ASOs across all gesture types (Figs 2–5). Fifteen gesture types were suitable for analysis, having been used by at least 3 individuals at least 3 times to achieve an ASO or ASOs (see Materials and methods for more information). If gestures were achieving outcomes at random, we would expect no difference between the distribution of a given gesture type and the average distribution across all gesture types. All but 1 gesture type (Object shake) showed significant deviation from the average distribution. Bonobo gesture types, like chimpanzee gesture types [28], do have distinct (sets of) meanings.

### Comparison of bonobo and chimpanzee gesture meanings

**Fig 6:**
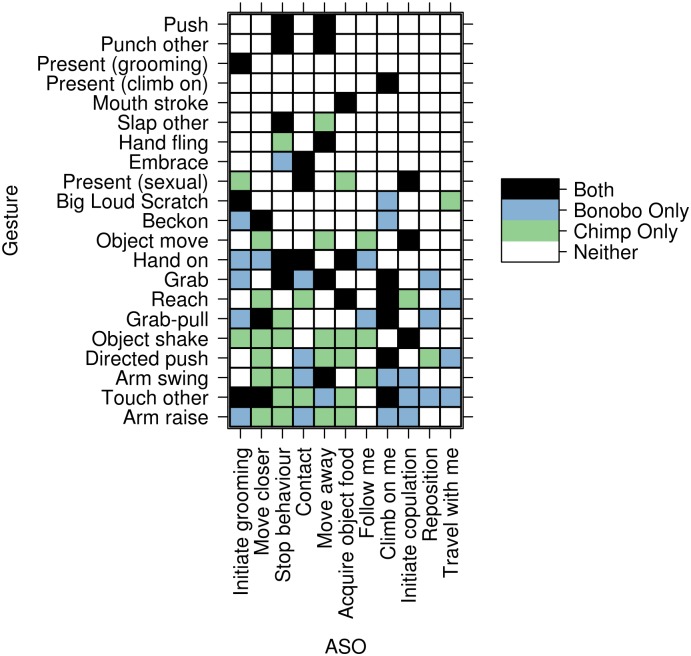
The overlap in gesture-to-ASO assignment between chimpanzees and bonobos (S1 and S2 Data). White cells correspond to gesture–ASO assignments absent in both species, green cells correspond to gesture–ASO assignments only present in chimpanzees, blue cells correspond to gesture–ASO assignments only present in bonobos, and black cells correspond to gesture–ASO assignments present in both species. ASO, Apparently Satisfactory Outcome.

**Fig 7:**
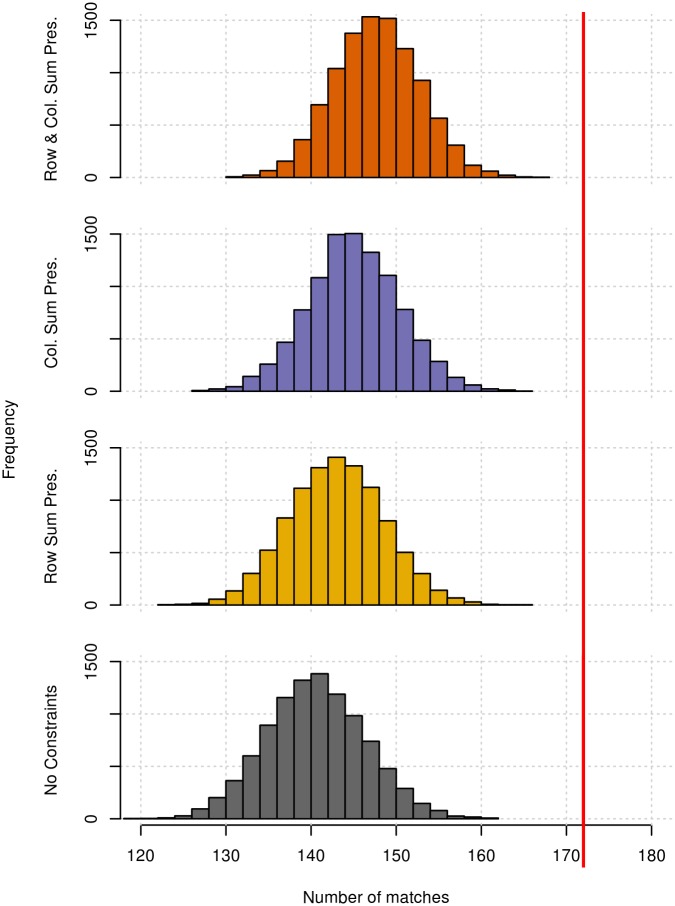
The frequencies of the total number of matches between species gesture matrices achieved by 10,000 iterations of the permutation test using 4 different constraints on matrix permutation (S1 and S2 Data). From bottom to top: unconstrained randomisation of assignments (No Constraints, grey), preservation of only the number of ASOs assigned to each gesture (Row Sum Pres., yellow), preservation of only the number of gestures assigned to each ASO (Col. Sum Pres., purple), and preservation of the number of gestures assigned to each ASO and the number of ASOs assigned to each gesture (Row & Col. Sum Pres., orange). The total number of matches in the original gesture matrices is given by the red vertical line. ASO, Apparently Satisfactory Outcome.

Using a randomisation procedure, we tested the null hypothesis that the similarity between the 2 species (Fig 6) would be the same under a random assignment of gestures to ASOs for each species (see Materials and methods). We compared 4 different methods of matrix permutation (R code in S1 Data), generating gesture-to-ASO assignment matrices with (a) no constraints (least conservative), (b) constraints on the column sums, (c) constraints on the row sums, and (d) constraints on both column and row sums (most conservative), none of which produced a pair of matrices that were more similar than the original data (Fig 7). When constraining the column or row sums, the total number of ASOs a gesture was assigned to (row sum preserve) or gestures an ASO was assigned to (col. sum preserve) in a permutation was constrained to that of the original chimpanzee and bonobo matrices, though the actual assignment is random. For example, under the row sum preserve method, the row “Object Shake” would have exactly 7 1s for any permutation of the chimpanzee matrix and a single 1 for the bonobo matrix, as in the original data (raw data and species matrices in S2 Data). We can be confident that the similarity of the gesture matrices for the 2 species is greater than expected by chance assignment of gestures to ASOs, as defined by the randomisation procedure.

**Fig 8:**
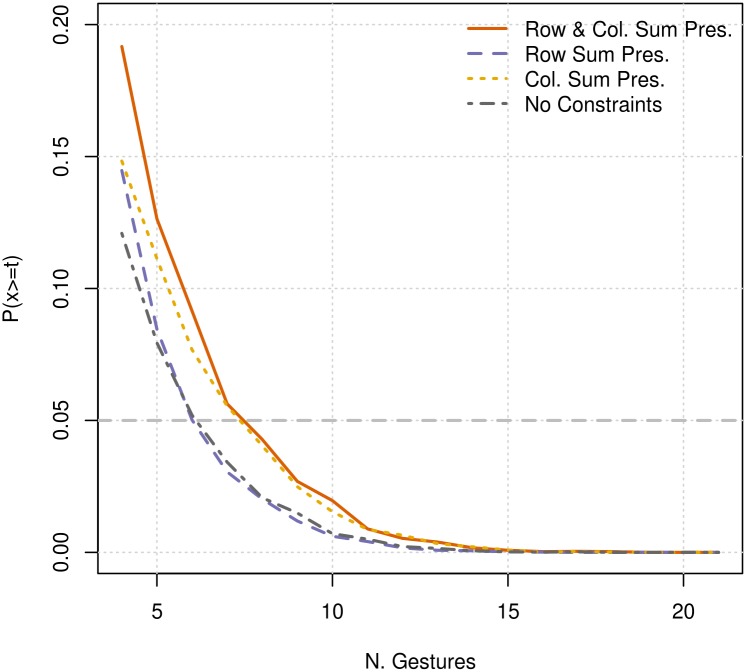
The probability of generating randomised gesture matrices that are more similar than those observed when a random subset of available gestures is used (S1 and S2 Data). Each line corresponds to 1 of the 4 different matrix randomisation constraints: preservation of the number of gestures assigned to each ASO and the number of ASOs assigned to each gesture (Row & Col. Sum Pres., orange), preservation of only the number of ASOs assigned to each gesture (Row Sum Pres., yellow), preservation of only the number of gestures assigned to each ASO (Col. Sum Pres., purple) and unconstrained randomisation of assignments (No Constraints, grey). ASO, Apparently Satisfactory Outcome.

We further explored the randomisation procedure, in order to find the limits of its application to communication, by repeating the randomisation process on subsets of our data. Specifically, we examined the effects of the available number of observed gestures on the results of our analysis by subsetting our original data to include an incrementally increasing number of gestures, from 4 to the maximum 21 available for use. The probability of generating randomised gesture matrices, using any of the 4 different constraint sets described above, that are more similar than the original gesture matrices remains very low (<0.05) as long as at least 8 or more of the gestures are included in the randomisation (Fig 8). The fact that such a strong signal of similarity between the 2 species’ gesture matrices exists with considerably less data lends weight to the robustness of our main result.

## Discussion

Bonobos intentionally deploy gestures to achieve at least 14 different intended outcomes—12 that initiate or develop an activity and 2 that stop it. They use gestures to request things (such as food) and to initiate co-locomotion, grooming, and sex. Because the gestures are intentionally produced, meeting widely accepted criteria for intentional communication [18], these outcomes are not only the gestures’ ‘functions’—they are their ‘meanings’ [20,28]. Moreover, bonobo gesture types have distinct (sets of) meanings. Almost all gesture types achieve a different distribution of ASOs to the average distribution, showing distinct aims. Object shake, the 1 exception, may have failed to show a distinctive pattern because of its small sample size and because it is primarily used for sex and grooming—2 behaviours that numerically dominate bonobo gesture instances and thus contribute substantially to the average distribution. Overall, we conclude that bonobos are using gestures to achieve distinct outcomes, as has also been found for chimpanzees [28]. About half of bonobo gestures have only a single meaning, while the others have 2 or more meanings. Words in human language can have a single meaning or polysemous meanings, and this poses no problem for the recipient in deciphering the signaller’s intended meaning. Future research (with an expansive enough dataset) can explore how bonobo recipients appear able to correctly interpret the meaning of apparently ambiguous gesture types, perhaps by including analysis of facial expressions, gesture sequences, or local situational context.

Having catalogued the meanings of bonobo gestures, we then compared them with the meanings of chimpanzee gestures, finding evidence of their similarity. Across 10,000 random permutations of the gesture matrices, we failed to generate a single pair that were more similar than the observed data, implying a negligible probability that such similarity arose by chance. This was the case even under the most conservative constraints, where randomised matrices were generated maintaining the number of assignments in both columns and rows as the original data. Our findings remained robust, with clear similarities found between bonobo and chimpanzee meanings, even with subsets of our main gesture matrices, down to a minimum of just 8 (of the 21 total) gesture types. In the future, researchers would ideally be able to use these methods to compare the meaning of gesture repertoires among a range of primate species, determining whether more closely related species have more similar gesture repertoires.

The bonobo and chimpanzee gestural repertoires—that is, the physical form of the gestures—overlap by 88%–96% [30]. We now know that there is also a large overlap in the intended outcomes achieved by these shared gesture types in bonobos and chimpanzees. Whilst biological inheritance is one possible explanation for this overlap, we recognise that similar gestures and meanings could emerge through another acquisition mechanism, such as ontogenetic ritualization [35] or a version of imitation [36,37] (but see [17]). Bonobos and chimpanzees also experience similar environmental and anatomical constraints that may restrict the available gestures and desired outcomes. More research is needed to explore the precise mechanism behind the overlap of gesture meanings. It is probable that this pattern of gestures and meanings also applied to the last common ancestor we shared with the 2 *Pan* species. That is, it is likely that the *Pan-Homo* last common ancestor would have been able to use and understand most of the gestures of modern bonobos and chimpanzees; less likely, but not impossible, the elaborate shared *Pan* repertoire could have evolved after divergence from the hominin lineage. If we can now discover whether humans also share or understand these great ape gestures, those 2 possibilities can be resolved [38]. Doubtless, gestural communication was an important contributor in the evolution of language [39,40]; but it remains to be seen how gesture as it manifests in nonhuman great apes relates to gesture as it manifests in humans alongside thought and language. Understanding this ‘baseline’ of gestural communication may better enable us to predict those new meanings that development of protolanguage offered to human-specific ancestors, ultimately resulting in the evolution of language.

## Materials and methods

### Subjects

KEG collected data on 2 neighbouring communities of wild bonobos (E1 group and P group) at Wamba, Luo Scientific Reserve, Democratic Republic of the Congo (00° 10' N, 22° 30' E). Habituation began for E1 group (*n* = 39) in 1976 (when it was still part of E group) and for PE group (*n* = 30) in 2010 (24, 25). At the beginning of this study, in 2014, the total sample size was 63 individuals, with 28 adults, 12 adolescents, 9 juveniles, and 14 infants. In 2015, the total sample size was 64 individuals, with 30 adults, 8 adolescents, 10 juveniles, and 16 infants. Bonobo age groups are divided into infant (<4 years), juvenile (4–7 years), adolescent (8–14 years), and adult (15+ years) [41].

CH collected data on 1 community of wild chimpanzees (Sonso community) at the Budongo Conservation Field Station, Uganda (1° 35’–1° 55’ N, 31° 18’–31° 42’ E). Habituation began for the Sonso community (*n* = 92) in 1990. At the beginning of this study, in 2007, the total sample size was 81 individuals, with 32 adults, 16 subadults, 15 juveniles, and 18 infants. Chimpanzee age groups are divided into infant (≤4 years), juvenile (5–9 years), subadult (male: 10–15 years, female: 10–14 years), and adult (male: 16+ years, female: 15+ years) [42].

### Data collection and video coding

KEG conducted fieldwork from 4 February 2014 to 28 June 2014 and 19 January 2015 to 13 June 2015, following bonobos daily from approximately 05:50 to approximately 12:00, with a weekly schedule of 4 days on and 1 day off. Observation time amounted to 204 days. CH conducted fieldwork from 25 October 2007 to 8 March 2008, 13 April 2008 to 1 January 2009, and 5 May 2009 to 8 August 2009, following chimpanzees daily from approximately 07:30 to approximately 16:30, with a weekly schedule of 3 days on, 1 day off, 3 days on, 2 days off. Observation time amounted to 266 days.

We filmed social interactions using focal behaviour sampling, where the focal behaviour was whenever 2 or more individuals approached within 5 m of each other. We chose this criterion to ensure that any gestures preceding social interactions were recorded. KEG recorded video footage with a Panasonic HDC-SD90 video camera, using the 3-second pre-record feature to increase the likelihood of catching the gestures in time. CH used a MiniDV tape using a Sony Handycam (DCR-HC-55). We imported video footage each day, labelled it, and entered it into a clip directory in FileMaker Pro.

Filemaker Pro was also used for video coding. Each gesture instance (that is, a single gesture) was coded in a separate sheet, with the following information: signaller, recipient, signaller age and sex, recipient age and sex, gesture type, part of sequence, audience checking, response waiting, persistence, recipient response, and ASO.

The signaller is the individual who produces the gesture, and the recipient is the individual who is the target of the gesture. The gesture type is a category comprising physically similar gesture instances, where gesture instances are grouped by body part and action. A complete list of gesture types is described in [20], with additional bonobo gesture types found in [19]. A sequence is a series of gesture instances separated by <1 s and produced by 1 individual. Audience checking, response waiting, and persistence are all criteria for intentionality. For audience checking, we reported whether or not the signaller turned to face the recipient; for response waiting, whether or not they paused for >1 s after gesturing; and for persistence, whether or not they continued to gesture. To be included in analyses, we required that each gesture instance meet at least 1 of these criterion for intentionality.

Recipient response was categorical: No response, ASO, Gesture (if the recipient responded with a gesture), or Unknown. To analyse meaning, we only used gesture instances where the recipient responded with an ASO. For a gesture to be assigned an ASO, we required that the recipient react to the gesture sequence with an ASO (that is, a response that satisfies the signaller shown by cessation of gesturing). ASO was then the specific outcome by the recipient.

KEG coded all the bonobo video footage and, to test interobserver reliability, CH coded 100 gesture instances for several of the aforementioned categories: gesture type, audience checking, persistence, and signaller apparently satisfied. We analysed Cohen’s kappa for interobserver reliability of these variables giving 0.87 (almost perfect), 0.56 (moderate), 0.70 (substantial), and 0.63 (substantial), respectively. CH coded all of the chimpanzee video footage; interobserver was conducted in their 2011 paper [31], with another experienced coder coding 50 gesture instances for directedness, recipient attentional state, and gesture type (Cohen’s kappa: directedness, κ = 0.69 [substantial]; recipient attentional state, κ = 0.63 [substantial]; gesture type, κ = 0.86 [almost perfect]).

### Analysis of bonobo gesture meaning

We recorded 4,256 intentionally produced gesture instances for wild bonobos, but we only analysed the 2,463 gesture instances (including those in sequences) that successfully achieved an ASO. We then excluded gestures used in play (231 instances), because including cases where gestures were used playfully would risk masking their normal meaning—the very nature of play means that gestures would be used playfully, not necessarily containing the same meaning they would otherwise. All statistical analyses were conducted in R 3.2.3.

In accordance with Hobaiter & Byrne 2014 [28], we used a series of ANOVAs to analyse whether the specific distribution of ASOs for a gesture type differed from the average distribution (the distribution of ASOs across all gesture instances). For direct comparability, we set the same parameters in our analyses to those used in their previous study [28]. To be included in parametric analyses, we required that each gesture type achieve an ASO at least 3 times (per individual) by at least 3 individuals (we analysed 1,896 gesture instances; 15 gesture types were suitable for this analysis, and 51 individuals contributed data). Then, we converted the number of instances a gesture type achieved any 1 ASO into a proportion of the total number of gesture instances in which an individual used that gesture type. We also calculated the average distribution by converting the number of instances in which all gesture instances achieved each ASO into a proportion of the total number of gesture instances. For values of 0 or 1, we converted them in accordance with Snedecor and Cochran (0 → 1/(4N) and 1 → 1-(1/(4N)), where N is the total number of instances for that gesture type) [43]. Finally, to calculate how the specific distribution deviated from the average distribution, we subtracted the average from the specific distribution. We then conducted the ANOVA with this resulting deviation as the dependent variable, ASO as the independent variable, and signaller identity as a random effect. *P* values of < 0.05 show that the deviation of the specific from the average distribution is significant.

### Randomisation procedure for bonobo–chimpanzee comparison

The relationships between gestures and ASOs for each species were represented as a matrix in which each row corresponded to a possible gesture and each column corresponded to 1 of the possible ASOs. A ‘1’ in this gesture matrix indicated that the associated gesture was observed to precede the associated ASO in the corresponding species. The criterion for inclusion was that a gesture type must achieve the given ASO at least 2 times and by a minimum of 2 individuals (that is, ape individual A uses it once and ape individual B uses it once). Note that criteria for the previous ANOVA were necessarily strict to meet the requirements for parametric analyses. In comparing the communication of 2 species that differ markedly in social behaviour, it is important not to mistake differences in the frequency of use of signals, which are to be expected, with genuine differences in communication system. To avoid that error, we deliberately adopted a looser criterion so that subtle differences in the bonobo and chimpanzee repertoires could be detected but not confused with spurious differences in usage frequency.

A ‘0’ in the matrix indicated that such an association was not observed. In order to deal with the few cases where we had insufficient data for 1 or other species, we defined the possible gestures as the intersection of all gestures used (*n* = 22) and possible ASOs as the intersection of all ASOs observed across both species (m = 11). Thus, the dimensions of the gesture matrices were the same for both species (*n* × m), and each row and column had at least one ‘1’. We defined the similarity between 2 gesture matrices simply as the sum of all matching corresponding matrix entries, be they 0 or 1.

Using a randomisation procedure, we tested the null hypothesis that the similarity between the 2 species would be the same under a random assignment of gestures to ASOs for each species [44]. To perform the randomisation test, we iteratively generated new gesture matrices for each species by randomly permuting the original gesture matrices and calculating the similarity between the 2 resultant matrices, generating a null distribution for similarity over 10,000 iterations. We used 4 different methods of permutation, each imposing different constraints on the possible matrices that could be generated. The simplest method simply randomly shuffled the values in each matrix without any constraints. The row sum preserve method shuffled the entries in each row of a matrix, thus preserving the number of ASOs assigned to each gesture for each species. The column sum preserve method shuffled the entries in each column, thus preserving the number of gestures allocated to each ASO. Finally, the row and column sum preserve method maintained both the number of gestures allocated to ASOs and ASOs allocated to gestures and was performed using the “tswap” algorithm in the vegan package in R, which implements a swap algorithm to generate new matrices that preserve row and column sums whilst sampling the distribution of possible matrices with equal probability [44]. From the null distribution of similarity values generated by each method, we calculated a corresponding *P* value as the proportion of iterations of the randomisation procedure in which the resultant similarity score was equal to or exceeded that of the original data.

We then examined the effects of the available number of observed gestures on the results of our analysis by subsetting our original data to include an incrementally increasing number of gestures, c, from 4 to the maximum 21 available to use. For each gesture count, c, and each iteration, we randomly sampled c gestures (rows) from both species’ gesture matrices to form the data subset for that iteration. Using this subset of the original data, randomised matrices were generated and the resultant similarity compared to that of the nonrandomised, subsetted matrices. A probability value was calculated as the proportion of iterations in which the randomised subset matrices were of equal or greater similarity than their nonrandomised subset counterparts. This probability value allowed us to test the null hypothesis that the observed similarity was the same as would be expected by chance, given that only c randomly selected gestures were observed. When c = 21, the maximum number of gestures available, any subset was the same as the original matrix, so the randomisation test was equivalent to that described in the main text.

It should be noted that this randomisation test does not rule out the possibility of another primate species having a more similar gesture–ASO matrix to chimpanzees or bonobos than they have to each other, though, to the best of our knowledge, no such data currently exist in order to test this. The procedure simply compares the similarity of the 2 species to the similarity of hypothetical gesture matrices generated under the above set of constraints.

## Supporting information

S1 Data R code for randomisation procedure. (R)

S2 Data Raw data and matrices for bonobo and chimpanzee gesture meanings. (XLSX)

S1 Table Values for Fig 1. By gesture type, the ASOs achieved and the number and proportion of instances that each ASO is achieved, ordered by the proportion for which the primary ASO is achieved (largest to smallest).
(DOCX)

S2 Table Individuals contributing data for ASO analysis. Number of individuals in each age and sex category that contributed data to analysis of ASOs for wild bonobos.
(DOCX)

## Abbreviations

ASO: Apparently Satisfactory Outcome
GG-rubbing: genito-genital rubbing
