# Supplementary material for: Bonobo and chimpanzee gestures overlap extensively in meaning
Source: PLoS Biol. 2018 Feb 27;16(2):e2004825. doi: 10.1371/journal.pbio.2004825 (PMC5828348; doi:10.1371/journal.pbio.2004825)
Supplement: S1 Table — By gesture type, the ASOs achieved and the number and proportion of instances that each ASO is achieved, ordered by the proportion for which the primary ASO is achieved (largest to smallest). (DOCX) [file pbio.2004825.s003.docx]

**S1 Table**. By gesture type, the ASOs achieved and the number and proportion of instances that each ASO is achieved, ordered by the proportion for which the primary ASO is achieved (largest to smallest).

| **Gesture type** | **Goal (ASO)** | **# Instances** | **Proportion** |
| --- | --- | --- | --- |
| Present (grooming) | Initiate grooming | 1063 | 1.00 |
| Big Loud Scratch | Initiate grooming | 67 | 1.00 |
| Directed push | Climb on me | 46 | 1.00 |
| Present (climb on) | Climb on me | 43 | 1.00 |
| Mouth stroke | Acquire object/food | 39 | 1.00 |
| Embrace | Contact | 11 | 1.00 |
| Leg flap | Initiate copulation | 8 | 1.00 |
| Hand fling | Move away | 8 | 1.00 |
| Bipedal rocking | Initiate GG-rubbing | 7 | 1.00 |
| Push | Move away | 7 | 1.00 |
| Beckon | Climb on me | 6 | 1.00 |
| Hip thrust | Initiate copulation | 5 | 1.00 |
| Stroking | Initiate GG-rubbing | 5 | 1.00 |
| Slap other | Stop behaviour | 4 | 1.00 |
| Tandem walk | Initiate grooming | 4 | 1.00 |
| Head rock | Initiate GG-rubbing | 3 | 1.00 |
| Roll over | Contact | 3 | 1.00 |
| Dangle | Initiate GG-rubbing | 15 | 0.79 |
|  | Initiate copulation | 4 | 0.21 |
| Arm up | Contact | 16 | 0.76 |
|  | Climb on me | 5 | 0.24 |
| Present (genitals forward) | Initiate GG-rubbing | 298 | 0.64 |
|  | Initiate copulation | 168 | 0.36 |
| Leg swing | Initiate copulation | 5 | 0.63 |
|  | Initiate GG-rubbing | 3 | 0.38 |
| Present (genitals backward) | Initiate copulation | 15 | 0.58 |
|  | Mount me | 11 | 0.42 |
| Reach | Climb on me | 19 | 0.58 |
|  | Acquire object/food | 7 | 0.21 |
|  | Climb on you | 7 | 0.21 |
| Punch other | Move away | 4 | 0.57 |
|  | Stop behaviour | 3 | 0.43 |
| Bipedal stance | Initiate GG-rubbing | 16 | 0.55 |
|  | Initiate copulation | 10 | 0.34 |
|  | Climb on you | 3 | 0.10 |
| Grab-pull | Follow me | 57 | 0.53 |
|  | Reposition | 33 | 0.31 |
|  | Climb on me | 8 | 0.07 |
|  | Move closer | 6 | 0.06 |
|  | Initiate grooming | 4 | 0.03 |
| Rocking | Initiate GG-rubbing | 16 | 0.52 |
|  | Initiate copulation | 12 | 0.39 |
|  | Contact | 3 | 0.10 |
| Object shake | Initiate GG-rubbing | 8 | 0.44 |
|  | Initiate copulation | 6 | 0.33 |
|  | Initiate grooming | 4 | 0.22 |
| Arm swing | Initiate copulation | 12 | 0.38 |
|  | Initiate GG-rubbing | 10 | 0.31 |
|  | Climb on me | 7 | 0.22 |
|  | Contact | 3 | 0.09 |
| Hand on | Contact | 7 | 0.35 |
|  | Initiate grooming | 4 | 0.20 |
|  | Climb on you | 3 | 0.15 |
|  | Follow me | 3 | 0.15 |
|  | Stop behaviour | 3 | 0.15 |
| Grab | Climb on me | 10 | 0.32 |
|  | Reposition | 8 | 0.26 |
|  | Initiate grooming | 5 | 0.16 |
|  | Stop behaviour | 5 | 0.16 |
|  | Contact | 3 | 0.10 |
| Arm raise | Initiate grooming | 25 | 0.31 |
|  | Initiate copulation | 17 | 0.21 |
|  | Initiate GG-rubbing | 16 | 0.20 |
|  | Climb on you | 15 | 0.19 |
|  | Climb on me | 4 | 0.05 |
|  | Contact | 4 | 0.05 |
| Touch other | Climb on me | 19 | 0.31 |
|  | Initiate GG-rubbing | 10 | 0.16 |
|  | Initiate grooming | 7 | 0.11 |
|  | Move away | 7 | 0.11 |
|  | Initiate copulation | 6 | 0.10 |
|  | Reposition | 5 | 0.08 |
|  | Stop behaviour | 5 | 0.08 |
|  | Travel with me | 3 | 0.05 |
