# Supplementary material for: Bonobo and chimpanzee gestures overlap extensively in meaning
Source: PLoS Biol. 2018 Feb 27;16(2):e2004825. doi: 10.1371/journal.pbio.2004825 (PMC5828348; doi:10.1371/journal.pbio.2004825)
Supplement: S2 Table — Number of individuals in each age and sex category that contributed data to analysis of ASOs for wild bonobos. (DOCX) [file pbio.2004825.s004.docx]

**S2 Table**. Number of individuals in each age and sex category that contributed data to analysis of ASOs for wild bonobos.

| **Gesture Type** | **Age** | **Sex** | **# Individuals** |
| --- | --- | --- | --- |
| Arm raise | Adult | Female | 2 |
|  | Adult | Male | 1 |
|  | Adolescent | Female | 2 |
|  | Adolescent | Male | 1 |
|  | Juvenile | Male | 2 |
|  | Infant | Female | 1 |
| Arm up | Adult | Female | 3 |
|  | Adult | Female | 6 |
|  | Adult | Male | 2 |
|  | Adolescent | Female | 1 |
|  | Adolescent | Male | 1 |
| Bipedal Stance | Adult | Female | 1 |
|  | Adolescent | Female | 1 |
|  | Adolescent | Male | 1 |
|  | Juvenile | Male | 1 |
| Directed Push | Adult | Female | 4 |
| Grab | Adult | Female | 3 |
|  | Adult | Male | 1 |
| Grab-pull | Adult | Female | 9 |
|  | Adult | Male | 2 |
|  | Adolescent | Female | 1 |
|  | Adolescent | Male | 1 |
| Mouth stroke | Adult | Female | 2 |
|  | Adolescent | Female | 1 |
|  | Juvenile | Male | 1 |
|  | Infant | Male | 1 |
| Object shake | Adult | Female | 1 |
|  | Adult | Male | 1 |
|  | Adolescent | Female | 1 |
| Present (climb on) | Adult | Female | 7 |
| Present (genitals forward) | Adult | Female | 19 |
|  | Adult | Male | 9 |
|  | Adolescent | Female | 3 |
|  | Adolescent | Male | 6 |
|  | Juvenile | Female | 3 |
|  | Juvenile | Male | 1 |
| Present (grooming) | Adult | Female | 18 |
|  | Adult | Male | 12 |
|  | Adolescent | Female | 5 |
|  | Adolescent | Male | 6 |
|  | Juvenile | Female | 4 |
|  | Juvenile | Male | 2 |
| Reach | Adult | Female | 4 |
|  | Infant | Male | 1 |
| Rocking | Adult | Female | 1 |
|  | Adult | Male | 2 |
| Touch other | Adult | Female | 6 |
|  | Adolescent | Female | 1 |
|  | Juvenile | Male | 1 |
